# Editorial: Neuromodulation by digital and analog drugs in consciousness research

**DOI:** 10.3389/fnsys.2023.1238151

**Published:** 2023-06-27

**Authors:** Darren F. Hight, Anthony G. Hudetz, Axel Hutt

**Affiliations:** ^1^Department of Anaesthesiology and Pain Medicine, Inselspital, Bern University Hospital, University of Bern, Bern, Switzerland; ^2^Department of Anesthesiology, Center for Consciousness Science, University of Michigan, Ann Arbor, MI, United States; ^3^Team MIMESIS, INRIA, Strasbourg, France; ^4^Team MLMS, UMR7357 Laboratoire des sciences de l'Ingénieur, de l'Informatique et de l'Imagerie (ICube), Illkirch, France; ^5^Team MLMS, University of Strasbourg, Strasbourg, France; ^6^Team MLMS, CNRS, Strasbourg, France

**Keywords:** aerobic exercise, fibromyalgia, anesthesia, information processing, criticality

The purpose of this Frontiers Research Topic was to enhance our scientific understanding of the relationship between different kinds of neuromodulation and their effects on states of consciousness. As such, the topic was deliberately broadly framed, and the diversity of the resulting publications keenly illustrates the breadth of the field of neurostimulation and consciousness.

Two contributions have focussed on non-pharmacological interventions. In the first, Cassim et al. investigated the effect of aerobic exercise on the sleep microstructure of older adults, as measured by a commercially available EEG sleep monitor. While aerobic exercise is not generally thought of as kind of neurostimulation, it is a highly accessible form of physical activity that has neuromodulatory effects besides the well-described endorphin rush often named “runners-high.” In this preliminary investigation, a gently increasing exercise intervention completed over 12 weeks led to a subsequent prolongation of deep sleep, as measured by an increase in EEG power in the slower frequency range, a difference associated with improved sleep quality. As the authors note, this exercise-induced shift was to some degree attenuating the usual EEG characteristics associated with increasing age.

In the second study also investigating a non-pharmacological intervention, Ramasawmy et al. examined if mindfulness meditation together with transcranial direct current stimulation might reduce levels of pain caused by fibromyalgia. This study had a double-blind and sham-controlled design, but did not find a difference in their main outcome of reported pain. Nonetheless, the authors did observe an increased level of quality of life in patients who had received current stimulation. It is important to note that both of the above studies are preliminary in nature, and preclude firm conclusions due to limited sample size.

Two further studies focused on anesthesia as a neuromodulator of states of consciousness. Shahaf et al. were motivated by a simple but insightful concern; namely, that if subsequently remembering an event that occurred during surgery (recall) is acceptable following sedation (where patients did not need to be “asleep” for their surgery), then a neuroimaging method that can detect recall may also be useful during general anesthesia (where patients *are* required to be “asleep”). The authors found that a new proposed measure of attention, the cognitive effort index, was higher in sedation cases with subsequent recall, than in patients without recall, and that this was more likely to occur in later stages of the case. The idea of being able to measure attention is very important, and future work will also need to distinguish between internally vs. externally directed orientation (Casey et al., 2022; Hight and Sleigh, 2022).

The second article focused on anesthesia was from Hutt and Hudetz who looked at the effects of electrical stimulation of a particular nucleus within the brainstem. This nucleus plays an important role in regulating arousal, and the authors investigated the effect of stimulation on levels of information processing and storage in rats. Interestingly, the effect of stimulation on these outcomes was largely context dependent. When analyzing slow waves, stimulation led to less information processing if anesthetic levels were low, but to more information processing if anesthetic levels were high. The authors interpreted this finding as support for the idea that the brainstem can act like a tuning amplifier on the cortex—if neuronal activity is mostly irregular (as with low anesthesia levels), brainstem function increases that irregularity, whereas if neurons are already highly synchronized (as during high anesthesia doses) stimulation will enhance this synchrony. While this effect was variable between individual animals, these findings will help to advance our theoretical and experimental knowledge of the relationship between electrical neurostimulation and brain state.

A final contribution from Gervais et al. is a timely scoping review on the relationship between the concept of criticality and altered states of consciousness. A system can be thought of as showing criticality when even a small perturbation may throw the system into a different state (e.g., “the straw that broke the camel's back”), and criticality has been associated with enhanced information processing during consciousness (O'Byrne and Jerbi, 2022). In their review, the authors surveyed a breadth of articles from many fields with a particular eye on where deviations from criticality might be associated with various disorders of consciousness. They find that deviations from criticality are associated with changes in consciousness in the literature, but also that there are diverse measures used, and that the literature in the field is very inhomogeneous.

We conclude from this collection of studies that pharmacological and non-pharmacological neuromodulatory applications influence states of consciousness, and show promise for the future. This may happen through influencing patient outcomes and may yield insights into the neural mechanisms of consciousness.

## Author contributions

DH drafted the manuscript. AH and AGH edited and approved the manuscript. All authors contributed to the article and approved the submitted version.
